# Clinical experience: Outcomes of mesenchymal stem cell transplantation in five stroke patients

**DOI:** 10.3389/fmed.2023.1051831

**Published:** 2023-01-19

**Authors:** Nesrin Ercelen, Nilgun Karasu, Bulent Kahyaoglu, Onder Cerezci, Rana Cagla Akduman, Defne Ercelen, Gizem Erturk, Gokay Gulay, Nagihan Alpaydin, Gizem Boyraz, Berrin Monteleone, Zekiye Kural, Hakan Silek, Sibel Temur, Canan Aykut Bingol

**Affiliations:** ^1^Department of Medical Genetics, Faculty of Medicine, Üsküdar University, Istanbul, Türkiye; ^2^Department of Neurology, American Hospital, Istanbul, Türkiye; ^3^Department of Physical Therapy and Rehabilitation, Faculty of Medicine, Üsküdar University, Istanbul, Türkiye; ^4^Department of Physical Medicine and Rehabilitation, American Hospital, Istanbul, Türkiye; ^5^Department of Neurology, Faculty of Medicine, Yeditepe University, Istanbul, Türkiye; ^6^Computational and Systems Biology Interdepartmental Program, University of California, Los Angeles, Los Angeles, CA, United States; ^7^Department of Healthcare Management, Faculty of Health Sciences, Üsküdar University, Istanbul, Türkiye; ^8^ATIGEN-CELL/Cell and Tissue Center, Trabzon, Türkiye; ^9^Geneis Genetic System Solutions, Istanbul, Türkiye; ^10^Department of Pediatrics at NYU Long Island School of Medicine, Medical Genetics, Langone Hospital, New York University, Long Island, NY, United States; ^11^Department of Anesthesia and Reanimation, Faculty of Medicine, Yeditepe University, Istanbul, Türkiye

**Keywords:** regenerative medicine, rehabilitation, stem cell therapy, stroke recovery, UC-MSC transplantation

## Abstract

Stem cell therapy, which has promising results in acute disorders such as stroke, supports treatment by providing rehabilitation in the chronic stage patients. In acute stroke, thrombolytic medical treatment protocols are clearly defined in neurologic emergencies, but in neurologic patients who miss the “thrombolytic treatment intervention window,” or in cases of hypoxic-ischemic encephalopathy, our hands are tied, and we are still unfortunately faced with hopeless clinical implementations. We consider mesenchymal stem cell therapy a viable option in these cases. In recent years, novel research has focused on neuro-stimulants and supportive and combined therapies for stroke. Currently, available treatment options are limited, and only certain patients are eligible for acute treatment. In the scope of our experience, five stroke patients were evaluated in this study, who was treated with a single dose of 1–2 × 10^6^ cells/kg allogenic umbilical cord-mesenchymal stem cells (UC-MSCs) with the official confirmation of the Turkish Ministry of Health Stem Cell Commission. The patients were followed up for 12 months, and clinical outcomes are recorded. NIH Stroke Scale/Scores (NIHSS) decreased significantly (*p* = 0.0310), and the Rivermead Assessment Scale (RMA) increased significantly (*p* = 0.0234) for all patients at the end of the follow-up. All the patients were followed up for 1 year within a rehabilitation program. Major clinical outcome improvements were observed in the overall clinical conditions of the UC-MSC treatment patients. We observed improvement in the patients’ upper extremity and muscle strength, spasticity, and fine motor functions. Considering recent studies in the literature together with our results, allogenic stem cell therapies are introduced as promising novel therapies in terms of their encouraging effects on physiological motor outcomes.

## 1. Introduction

A stroke is defined as a rapidly developing cerebrovascular disorder resulting in focal or global neurological deficits lasting more than 24 h (1). Stroke carries a global burden as the second most common reason for death and disability in the world (2). One out of every six people is affected by this condition worldwide (3). Over 13 million people experience strokes every year, and approximately 5.5 million die because of disease complications (4). According to current data, the incidence rate of stroke varies between high and low-income countries worldwide (5, 6). Although it more commonly affects the elderly population, recent studies emphasize the increased incidence of stroke in young individuals; hence, it becomes a major healthcare and socioeconomic issue for the entire population (7).

In ischemic stroke, occlusion of the cerebral arteries leads to ischemia and infarction of the brain (4). An ischemic stroke occurs when blood flow to an area of the brain is reduced under a critical threshold (8). This ischemic core is surrounded by hypo-perfused tissue with some preserved metabolic activities, which is also known as the penumbra. Penumbra denotes the potentially salvageable brain tissue amenable to reperfusion therapy (9). Hemorrhagic stroke is responsible for 10–15 percent of all strokes and has a high mortality rate. Hemorrhagic stroke occurs when blood vessels rupture, causing bleeding into the brain. Hemorrhagic stroke is relevant to a high rate of morbidity and mortality, and the most prevalent kind is intracerebral hemorrhage (10). Based on the degree and severity of the vascular dysfunction, stroke could lead to various deficits including but not limited to motor, sensory, and cognitive disturbances, and cranial neuropathies (11).

Current treatments mainly focus on saving the penumbra region by early vascular recanalization and thrombolysis, achieved in a narrow therapeutic window indicating the first 24 h following stroke onset. Most of the research focuses on this traditional early intervention approach aiming to improve cerebral blood flow and recanalization (11, 12). A blood clot obstructing a cerebral artery remains the leading cause of strokes. Immediate thrombolytic therapy can assist recovery after a stroke in some patients by recovering blood flow before significant brain impairment has formed. Thus, thrombolytic medications might create serious brain hemorrhages that can be fatal (13). On the other hand, recent studies began to point out that inflammation is also a significant component of ischemic stroke (14). Stroke creates an inflammatory microenvironment in the brain parenchyma (15). Hence, this neuroinflammatory process, which is the hallmark of stroke, could also act as a potential target for novel stroke therapies (16).

In the acute stages of stroke, after the initial ischemic insult, neurons and astrocytes produce damage-associated molecular patterns. Also, they promote a proinflammatory response and lead to the production of pro-inflammatory cytokines, such as TNF-α, IL-1, IL-6, followed by activation of M1 microglia and endothelial damage (17). The primary origin of the inflammatory cascade in brain injury is microglia (18). This results in blood-brain barrier (BBB) breakdown, further contribution to this pro-inflammatory microenvironment, allowing peripheral leukocytes to pass through BBB (17). Increased permeability of the blood-brain barrier worsens cerebral edema and exacerbates inflammatory damage, raising the post-stroke risk of bleeding (19). Recent studies pointed out that mesenchymal stem cells (MSC) could be used to revert this process, providing an anti-inflammatory environment that would enhance neurogenesis and angiogenesis, and hence, they may act as a potential treatment option for stroke in the subacute stages. MSC treatment can act by suppressing pro-inflammatory microglia M1 activity and increasing anti-inflammatory microglia M2 activity (18). In the study conducted in 2010, it was demonstrated that when MSCs transplanted *via* intracerebral or intravenously in animals with stroke models, MSCs can differentiate into neurons or glial cells, which means they could potentially replace damaged cells in the brain (20, 21). It has been shown that MSCs exhibit prominent immunomodulator, angiogenic, neurogenic, and regenerative effects. They are collectively named secretomes and are capable of secreting various paracrine bioactive molecules, such as cytokines, chemokines, growth factors, and proteasomes (22). Immunomodulation by MSCs is achieved by a combination of processes including cell-cell interaction, secretable substances, and crosstalk between signaling cascades. Also, in recent studies, the same effect is experienced by exosomes, small membrane vesicles containing bioactive substances, such as proteins, lipids, mRNAs, microRNAs, and mitochondria, which are secreted by MSCs and can connect with adjacent cells (10). MSCs exert different effects in the time interval of the administration. In fact, within the first two weeks of administration, MSCs create an anti-inflammatory environment where after a couple of weeks, MSCs begin to promote angiogenesis and neurogenesis, playing a role both in acute and chronic phases of stroke (22). Steinberg et al. reported in their open-label, single-arm, phase 1/2a study that the treatment of chronic stroke patients with modified bone marrow-derived mesenchymal stem cells (SB623) is safe. In their study, significant improvements in clinical outcomes are also declared (23).

Due to its excellent properties, such as simple isolation, multipotent differentiation potential, and powerful paracrine activity, MSC-based therapy has appeared as a novel strategy in recent decades (24). Due to all these unique characteristics, MSC transplantation is a promising treatment option for the stroke patients. The mode of delivery is just as crucial as the MSC application timing and dose. When administered intraventricularly, MSCs are disseminated over a wider range of cerebral regions, however, cell quantity is crucial in this case (25). On the other hand, when MSCs are administered intravenously, it is stated that MSCs are removed from the body within 24 h. However, due to the polarization of macrophages *via* cytokines, phagocytosis allows lysed transplanted cells to enter the bloodstream and to be transferred to many parts of the body *via* signaling pathways, possibly paracrine signals, and provides neuroprotection with a systemic effect (Figure 3; 26). Since the entire extent of stroke recovery has yet to be discovered, MSCs are highly amenable to the development of novel therapies (27).

In the present study, we aimed to investigate the therapeutic effects of mesenchymal stem cell transplantation in five patients with a history of stroke. Furthermore, our goal was to examine the clinical effects of immune modulation, anti-inflammatory, and neovascularization impacts of mesenchymal stem cells in stroke both in the acute and chronic periods.

## 2. Materials and methods

The study was conducted within the scope of clinical trials of tissue, cells, and advanced medical treatment products with the permission of the Ministry of Health of the Republic of Turkey. All the patient’s clinical files were submitted to the Ministry of Health Stem Cell Commission and approved. The signed information and consent forms of the patients’ families for the MSC treatment were included in the clinical files. The patients were enrolled in the study by the Declaration of Helsinki. 5 patients aged between 46 and 70 with a history of stroke were enrolled in the study. Patient demographics, comorbid conditions, and associated motor deficits are given in Table 1. Patients were initially assessed and followed up for 1 year by using the NIH Stroke Scale/Score (NIHSS), Rivermead Assessment Scale (RMA), Berg Balance Scale (BBS), and Chedoke Arm and Hand Activity (CAHAI-13) scores (Table 4; 28–31). Clinical progress was recorded after every visit in the 1st, 3rd, and 12th months during the follow-up.

**TABLE 1:** Patients’ demographic data.

No.	Age	Sex	Weight	Co-morbid condition	Diagnosis/Motor deficit
P1	46	Male	70	Hypertension	Hemiparesis (R)
P2	60	Female	60	Hypertension	Hemiplegia (L)
P3	67	Male	95	Hypertension, insulin resistance	Hemiparesis (L)
P4	53	Male	120	Diabetes mellitus	Quadriplegia
P5	70	Female	71,6	Hypertension, diabetes mellitus	Quadriplegia

Umbilical cord-mesenchymal stem cells (UC-MSCs) were administered to five patients with a history of stroke within the last 8 months (Table 5). Each patient received a single course of 1–2 × 10^6^ of Aticell-CH-MKH Vial Isolen injected into 100 ml of normal saline administrated with a rate of 50 ml/h.

The inclusion and exclusion criteria of patients included the following.

Inclusion criteria:

1. Consistent with recognized diagnostic criteria, with BT or MRI supporting the diagnosis.
2. Stable vital signs, including less than 220/120 mm Hg in the systolic blood pressure, 40–100 beats per minute in the heartbeat, less than 38.5° in the body temperature, and more than 92% in the oxygen saturation.
3. No contraindication for mobilization within 48 h of stroke onset.
4. Age under 70 years.
5. Ability and willingness to give informed consent.

Exclusion criteria:

1. Who received intravenous thrombolysis or arterial thrombectomy prior to admission. Since blood thinners (anticoagulants and antiaggregant) are used by stroke patients, aPTT and Pt values were investigated as a means of control.
2. Those with serious cardiovascular disease, bacterial and/or yeast secondary infection, uremia, hematological malignancies or diseases such as malignant tumors, cancer, and progressive neurodegenerative diseases such as dementia, Parkinson’s disease, and epilepsy.
3. With severe cognitive impairment, aphasia, or visual or hearing impairment that precludes assessment.

5 patients aged between 46 and 70 with a history of stroke within the last 8 months were enrolled in the study.

### 2.1. UC–MSC culturing

**FIGURE 1:**
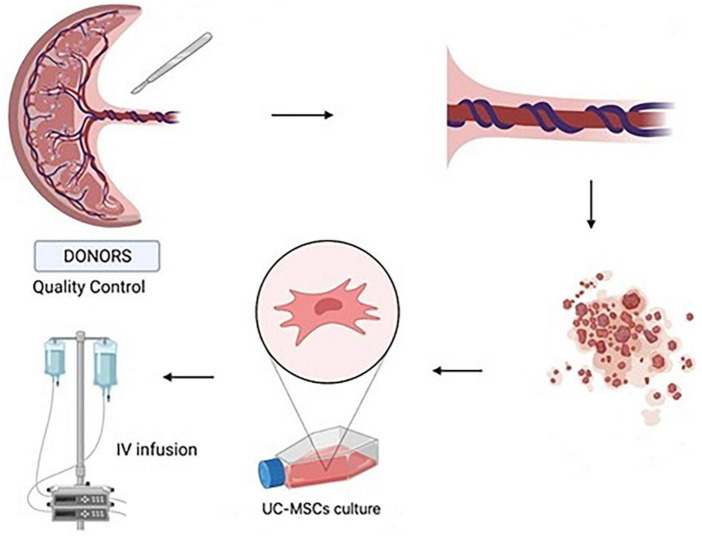
Umbilical cord tissue was used to isolate umbilical cord-mesenchymal stem cells (UC-MSCs) by primary culture. UC-MSCs obtained from the placenta were dissected into 1–2 mm pieces. They were cultured and amplified. The cell suspensions are administered intravenously to the patients (Created with BioRender.com).

**FIGURE 2:**
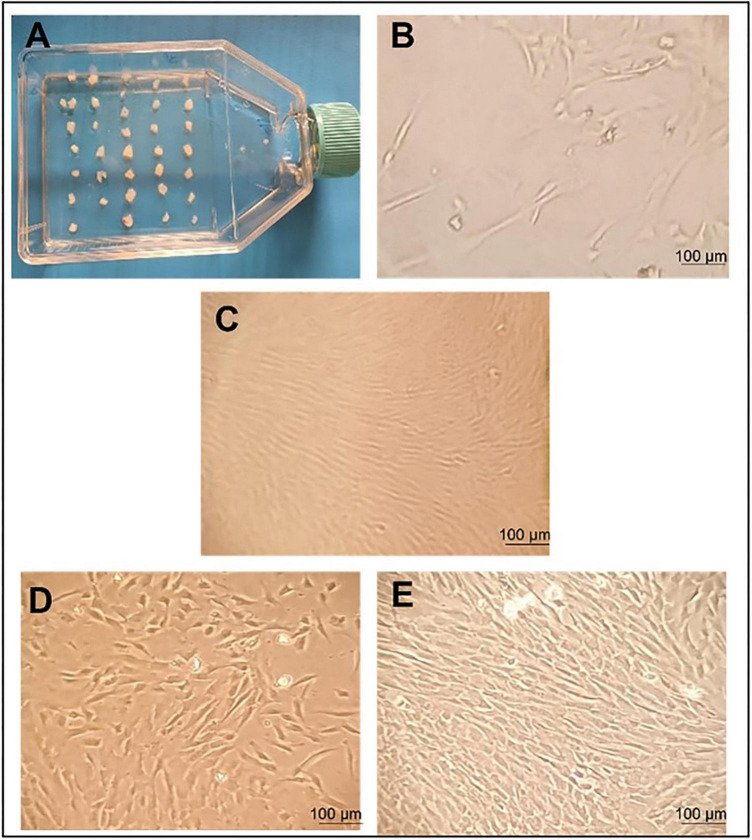
Umbilical cord tissue mesenchymal stem cells morphology and proliferation. Morphology of human mesenchymal stem cells (MSCs) observed using an inverted microscope. **(A)** initial day; incubation of 1–2 mm tissue in a flask. **(B)** MSC grows out from tissue pieces 10 days after seeding. Fusiform cells began to form around the tissue and colonization was observed. **(C)** Growing colonies; a substantial number of cells exhibited a fibroblast-like morphology 14 days after seeding. **(D)** The proliferation of fibroblastic cells 18 days after seeding. **(E)** The culture showed 80% confluent colonies 21 days after seeding. This is the image that shows the need to be passed to the P2 stage. Scale bars 100 μm.

UC–MSCs were isolated and proliferated according to good manufacturing practice (GMP) protocols with animal component-free media, quality assurance, and quality control for use in clinical applications (Figure 1). The clinical-grade MSCs were provided by the Turkish Health Ministry-licensed Atigen-Cell Technology Center in Trabzon, Turkey. A GMP standard methodology for UC-MSC isolation and culture was devised in this study (Table 2). The UC tissues are cut into pieces of 1–2 mm^3^. The explant method is used to inoculate into the flask. The smashed tissue is cultured in DMEM/F12 medium containing 10% serum, at 37°C in 5% CO2, and cell development is monitored. An upper passage process begins when the cells that surround the tissue develop. The passage process is repeated until the desired number of cells has been reached. The final product evaluation includes cell counting, viability, flow cytometry analysis, sterility, and endotoxin analyses of the ready cells (Figure 2; 32).

**TABLE 2:** Production of the clinical grade mesenchymal stem cells from umbilical cord tissue in good manufacturing practice (GMP) laboratory.

Wash the umbilical tissue three times with normal saline ↓
Divide the tissue into 2 cm pieces ↓
Remove 2 arteries 1 vein ↓
Cut the tissue into 1–2 mm^3^ pieces ↓
Place in flasks with culture method ↓
Follow up ↓
Follow the cellular structures around the tissue, passage when confluent ↓
Freeze/Send

**FIGURE 3:**
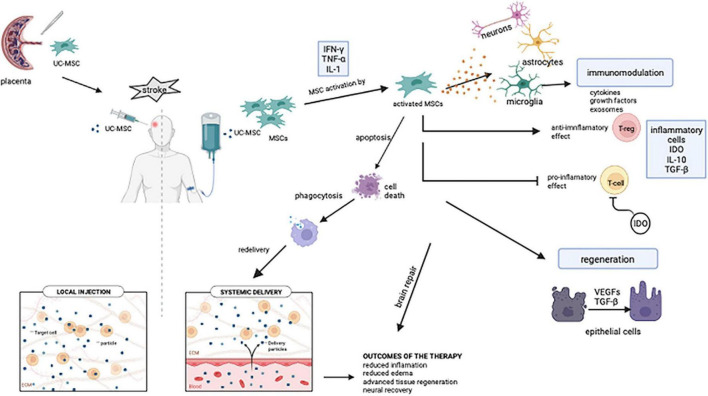
Intravenous MSC-mediated immunomodulation and tissue recovery potential mechanisms. Stroke patients received intravenous injections of mesenchymal stem cells obtained from the umbilical cord. Pro-inflammatory factors such as IL-1, IFNγ, and TNF-α facilitate MSC activation. Microglia, which are activated after MSCs are transplanted, contribute to the neuro repair of the brain by inducing an inflammatory response. IDO, which is expressed from MSCs, inhibits T cell growth. MSCs modulate the immune system with cytokines, growth factors, and exosomes. MSCs, respond to their surroundings by producing growth and differentiation factors and contributes to tissue remodeling after transplantation. Despite intravenously transplanted cells having a short half-life, their cellular residues can be phagocytosed and transported to various parts of the body *via* the bloodstream (UC-MSC, umbilical cord mesenchymal stem cell; MSC, mesenchymal stem cell; IFN-γ, interferon-gamma; TNF-α, tumor necrosis factor-α; IL-1, IL-10, interleukin family; IDO, indoleamine 2,3-dioxygenase; T-reg, regulatory T cell; TGF-β, transforming growth factor beta; VEGFs, vascular endothelial growth factors). Created with BioRender.com.

### 2.2. Statistical analysis

Statistical analysis was carried out in the GraphPad Prism 8.0.2 program. The normality of the group was determined by the Shapiro-Wilk test. To compare all patients’ pre- and post-treatment (at the end of the follow-up) NIHSS, RMA, and BBS scores, a parametric Paired Sample *T*-Test was performed, and *p* < 0.05 was considered statistically significant. Non-parametric Wilcoxon test was used to analyze the CAHAI-13 score.

## 3. Results

The route, dose, and cell type of MSCs administered as well as motor improvement, assessment modality, and major outcome results in 5 patients are summarized in Table 3. There were significant improvements in spasticity and motor functions in 5 patients in the first 12 months of follow-up. The NIHSS score decreased significantly (*p* = 0.0310), and the Rivermead Assessment Scale (RMA) scores increased significantly (*p* = 0.0234) in all 5 patients at the end of the 12 months. Despite this, no significant change was detected in BBS and CAHAI-13 scores (Table 4). The outcome measures in stroke rehabilitation are given in Table 4, and NIHSS, RMA, BBS, CAHAI-13 scales available at the end of the follow-up are given in Tables 3, 4. In four out of five patients, no infusion-related toxicities, fatalities, or severe adverse events could be associated with UC-MSC treatment. Patient P3 showed an acute allergic reaction, which was treated with Ketotifen 1 mg tablet and cetirizine 10 mg tablet. Pheniramine maleate 45.5 mg/2 ml was injected into isotonic 100 ml and administered to the patient.

**TABLE 3:** Clinical outcome of umbilical cord-mesenchymal stem cells (UC-MSC) treatment for stroke.

No.	Route	Dose	Cell type	Motor improvement	Assessment modality	Major outcome
P1	Intravenous	1–2 × 10^6^/kg	Allogenic UC-MSCs	Major	NIHSS, RMA, BBS, CAHAI-13	Improvement in hand and upper extremity functions
P2	Intravenous	1–2 × 10^6^/kg	Allogenic UC-MSCs	Major	NIHSS	Improvement in upper and lower extremity functions
P3	Intravenous	1–2 × 10^6^/kg	Allogenic UC-MSCs	Major	NIHSS, RMA, BSS, CAHAI-13	Improvement upper extremity functions
P4	Intravenous	1–2 × 10^6^/kg	Allogenic UC-MSCs	Major	NIHSS	Improvement in fine motor functions
P5	Intravenous	1–2 × 106/kg	Allogenic UC-MSCs	Major	NIHSS	Improvement in all extremity muscle strength

**TABLE 4:** Outcome measures in stroke rehabilitation after umbilical cord-mesenchymal stem cells (UC-MSC) treatment.

No.	NIHSS before treatment	NIHSS			RMA before treatment	RMA			BBS before treatment	BBS			CAHAI-13 before treatment	CAHAI-13		
		**After 1 month**	**After 3 month**	**After 12 month**		**After 1 month**	**After 3 month**	**After 12 month**		**After 1 month**	**After 3 month**	**After 12 month**		**After 1 month**	**After 3 month**	**After 12 month**
P1	10	7 (2nd day)	0	0	21	32	-	35	49	53	-	54	64	91	-	91
P2	18	6	6	6	6	8	-	14	0	7	-	20	13	14	-	19
P3	4	3	3	3	12	16	-	22	29	38	-	49	13	13	-	26
P4	38	30	30	30	0	0	-	0	0	0	-	0	13	13	-	13
P5	20	17	-	17	0	9	-	14	-	-	-	-	-	-	-	-

NIHSS, NIH stroke scale/score (*p* = 0.0310). RMA, rivermead assessment scale (*p* = 0.0234). BBS, berg balance scale. CAHAI-13, Chedoke Arm and Hand Activity.

**TABLE 5:** Mesenchymal stem cells (MSCs) transplantation information according to patients’ stroke history.

Patient	Stroke type	MSC therapy after stroke	MSC therapy outcomes
P1	Hemorrhagic	7 months	Major
P2	Ischemic	2 weeks	Major
P3	Ischemic	4 months	Major
P4	Hypoxic/Ischemic encephalopathy	5 months	Major
P5	Ischemic	1 year	Major

### 3.1. Clinical case progress

We evaluated the efficacy outcomes of the five UC-MSC transplanted patients as below.

#### 3.1.1. First case

A 46-year-old man was admitted to the emergency department with a complaint of cough, weakness, and back pain. His medical history is remarkable for hypertension. He was diagnosed with SARS-CoV-2 pneumonia and admitted to an inpatient clinic. He was discharged 4 days later and 1 week after his discharge his general condition rapidly deteriorated. He developed right-sided weakness both in his arm and leg. His cranial MRI revealed a left thalamic hemorrhage. The patient who received stroke treatment was discharged after 1.5 months with weakness on the left side.

The patient received MSC transplantation after 7 months after the stroke event (Table 5). He showed improvement in fine motor movements of the right hand, which became evident 4 weeks after the stem cell treatment. Rehabilitation continued in the Physical Therapy department. Major changes were observed in the patient’s right hand and upper extremity, the patient’s NIHSS scale decreased from 10 to 7 in 4 weeks, RMA increased from 21 to 32, Berg Balance Scale test from 49 to 53, and CAHAI-13 Test from 64 to 91 performed 3 months after the treatment (Table 4).

#### 3.1.2. Second case

A 60-year-old female patient was admitted to the hospital with a complaint of weakness on the left side. The preliminary diagnosis was ischemic cerebrovascular disease. In the examinations performed, the patient was diagnosed with left hemiplegia. She was followed with regular ischemic stroke treatment in the intensive care unit. With the family’s consent, the patient received MSC transplantation in the second week after the stroke. Significant improvement in the upper extremity functions was followed in the first year, while the NIHSS scale decreased from 18 to 6 in 4 weeks after the therapy. The loss on the left face completely recovered, the patient started oral feeding, and at the end of the first year, the swallowing reflex returned, the left-hand grip was restored, and there was little limp in the left leg (Table 4).

#### 3.1.3. Third case

A 67-year-old male patient developed left hemiparesis as a result of ischemic cerebrovascular disease on November 20, 2020. He was hospitalized and included in the neurological follow-up, physical therapy, and rehabilitation program. The patient has hypertension and insulin resistance, and the diagnosis of the patient was left hemiplegia. The patient was admitted to our clinic for UC-MSC treatment with an NIHSS score of 4. After the stem cell transplantation, 4 months after the patient’s standing safety and time have increased within a 5-week rehabilitation program (Table 5). The patient’s transition from standing to sitting position has increased to the point of being able to do it comfortably with help from the right arm. He can stand up without assistance, he can go from a lying position to a sitting position on the side of the bed without assistance, and he can climb 8–10 steps without help. Major changes were observed in the patient’s left hand and upper extremity, and the patient’s NIHSS scale decreased from 4 to 3 in 4 weeks after the stem cell treatment (Table 4). RMA increased from 12 to 16, the BBS test from 29 to 38 in the third month, and the CAHAI-13 test remained constant at 13 (Table 4).

#### 3.1.4. Fourth case

53-year-old male patient. The patient has no known diabetes or heart disease. He had an arrest and was intubated after CPR in an emergency center. The patient was admitted to our clinic for UC-MSC therapy in the 5th month after the stroke with the family’s consent (Table 5). NIHSS scale decreased from 38 to 30 after the therapy in 4 weeks. Although the NIHSS score remains to consult as 30 in 1 year after the therapy, we observed clinical improvement in fine motor functions.

#### 3.1.5. Fifth case

A 70-year-old female patient was brought to the hospital with the inability to breathe and weakness in the arms and legs. She was quadriplegic due to an acute pons infarction 1 year ago and was followed up as a home care patient. The patient has hypertension and Type 2 diabetes. Intravenous mesenchymal stem cell transplantation was applied to the patient 1 year after the stroke with the family’s consent (Table 5). As we signed on NIHSS before and after scores, she had a significant improvement in muscle strength after stem cell transplantation (Table 4).

## 4. Discussion

Stroke remains one of the top reasons for disability and mortality worldwide despite recent advances in novel therapy options (33). In many major clinical studies, a variety of pharmacological interventions have been tested to diminish tissue damage and cultivate functional outcomes after stroke, however, the targeted effects have not been achieved yet (34). Despite all the advances in science, the treatment options available for stroke are still confined. Early reperfusion is an intravenous therapy that works best if applied within the first 4–5 h after a stroke, but it is confined to patients with proximal occlusion and facilities with private treatment options such as mechanical thrombectomy (35). Intravenous thrombolysis has been regularly utilized for more than 20 years, but treatment delays continue to be a serious issue. The biggest lag in thrombolysis administration is due to pre-hospital variables. Also, assessment, imaging, and thrombolysis administration all play a role in lagging. If thrombolytic therapy is postponed, the chances of improvement of the patient’s functional state are reduced, and the potential risk of hemorrhage raises. Thus, to get the most out of thrombolysis, more integrated procedures are needed (36). Hence, all these options are insufficient to treat stroke patients, and no effective treatment has been obtained yet (37). Since there are still millions of affected individuals, a prompt and urgent response is required for stroke. It is challenging to take advantage of neurogenesis due to the intricate interplay of elements related to neuroinflammation. At this point, MSCs appear to be quite effective and have a bright future (16). Only by comparing data from numerous researches including *in vitro*, *in vivo*, and clinical experience, can this complex process be comprehended.

Our research provides significant data in terms of the clinical outcomes of mesenchymal stem cell therapy, which will provide hope for patients suffering from a stroke. While there were significant changes in the data acquired from 5 patients with NIHSS and RMA scores, there were no significant changes in the data obtained from 4 patients with BBS and CHAI-13 scores. According to these findings, neurologic recovery can be attained to a considerable extent following MSC treatment, while physical rehabilitation requires more time. With multiple mechanisms that can effectively overcome the pathophysiological changes following stroke, stem cell therapy became a novel therapeutic strategy for curing stroke. In the literature, cells derived from umbilical cord blood (UCB) offer various upsides for biological therapy in stroke (38). UC-MSCs can be acquired in a non-invasive way and have a high plasticity potential as well as regenerative, immunomodulatory, and immunotolerance properties (39–41). Laskowitz et al. reported that in 2018, infusing allogenic UCB cells in ischemic stroke patients would develop amelioration by reducing inflammation and supporting neuroprotection and plasticity. In conclusion, they stated that there were fewer neurological, physical, and functional deficiencies in stroke patients after UC-MSC treatment indicating UC-MSC immunomodulation effect in acute ischemic stroke (3). Stem cell therapy, which has been demonstrated to be effective in the treatment of acute diseases, improves treatment effectiveness by allowing rehabilitation during the disease’s chronic phase (23, 42, 43).

Since there is now no proven therapeutic treatment for stroke patients, MSCs appear miraculous in the treatment of stroke. Our study contributes to the body of knowledge on stroke patients’ improved motor function. It is important to note in the literature the tremendous improvement we observed in patients. On the other hand, bone marrow-derived cells are frequently used in research. However, in our study, umbilical cord derived MSCs were used, which have a higher plasticity capacity, a faster rate of cell division, and it may be acquired non-invasively in high concentrations. The most common method of administering therapeutic MSCs now is intravenous injection (44). Moreover, the safe use of allogeneic stem cells in place of autologous ones and the utilization of the quick and painless intravenous route for administration make our research significant.

Mesenchymal stem cells (MSCs) stem cells can be derived from different tissues (45). Bone marrow, umbilical cord blood, and umbilical cord tissue include cells competent for derivation down the hematopoietic lineage. Preclinical data shows that both bone marrow and umbilical cord blood cells can be given to animals with stroke and have a positive impact on neurologic healing (11). In stroke, neuronal loss occurs when small vessels in the brain are occluded. This neuronal damage triggers the inflammatory response cascade. The study with an animal stroke model showed that the damaged area shrunk significantly with direct injection of MSCs into the brain or intravenous MSCs administration. It has been stated that despite the MSC therapy ultimately providing neuronal recovery, the mechanism behind MSC-induced functional recovery is still obscure (46). In the literature, it was indicated that the route of delivery, dose, number of injections, and tissue from which MSCs originate are all factors that influence the success of MSC therapy, but it is still unclear which one is the most supportive of the actual recovery (47).

In recent studies, it was evaluated that MSCs reduce inflammation *via* immunomodulation with its effect at the peripheral level. Angiogenesis, astrocytes, neurogenesis, axons, and oligodendrocytes play a role at the central level (27). The immune response is significant in the pathogenesis of ischemic stroke because it is initially necessary to limit ischemic stress. Following a stroke, innate immunity is immediately activated and stimulates inflammation. Adaptive immunity is also triggered by the increase of inflammatory mediators. However, the sustained and unrestricted inflammatory response can cause major injury to the penumbra after the local brain injury. In this case, regression of post-stroke inflammation and repair of neural injury have life-saving importance (27). When tissue is injured due to stroke, an inflammation process commences, and MSCs move to the damaged scene (48). The therapeutic mechanism of action of MSCs is thought to be based on the creation of an anti-inflammatory and regenerative microenvironment on the damaged tissue surrounded by inflammatory cytokines in the presence of severe inflammation (Figure 3). Also, hypoxia and extracellular matrix components affect the immunomodulatory activity of MSCs. In a model of myocardial infarction, it was demonstrated that MSCs in hypoxic conditions secrete factors like IL-6, vascular endothelial growth factor (VEGF), and chemokines resulting in enhancing the therapeutic effects of MSCs (49). It is unanimously stated in recent studies that MSCs require a particular threshold of inflammation for immunosuppressive capabilities. It has even been shown that the efficacy of treatment decreases when MSCs are injected before the onset of inflammation (48). Inflammatory factors such as IL-17A, IFNy, and TNF, can mediate pro-inflammatory responses that facilitate MSC activation, contribute to MSC immunosuppression, and hence, heal. MSCs express indoleamine 2,3-dioxygenase (IDO), which inhibits T cell growth and activity in their surroundings. Overall, MSCs regulate the immune system by producing cytokines, growth factors, anti-inflammatory mediators, and exosomes (49).

Mesenchymal stem cells (MSCs), according to the literature, are removed quickly after injection, yet their therapeutic effects maintain despite this. MSCs injected intravenously act on the injured area *via* paracrine signaling and cell-cell interaction, regulate immune cell function, and suppress inflammation through the induction of T-reg cells (40, 48, 50). In a study in which different administration routes were investigated in non-obese diabetic mice for MSC transplantation *via* fluorescent signaling by an *in vivo* imaging system, they stated that MSCs induce caspase 3-mediated apoptosis and are swallowed by macrophages quickly after transplantation (44). Besides, in the study in 2018, they explored the process of the transplanted MSC and their immunomodulatory effects following application, demonstrating that infused MSC are swiftly removed through monocyte phagocytosis. When monocytes differentiate into macrophages, they can trigger adaptive immune responses (51). In the study, Wang et al. determined that MSCs diminished the expression of nuclear factor kappa B (NF-kB) *via* the VEGF signaling pathway. NF-kB inhibition is associated with the anti-inflammatory and anti-apoptotic effects of MSCs (27). In literature, it has been emphasized that many factors provide neural recovery following MSC therapy in stroke and that astrocytes, which especially promote brain plasticity, are significant in terms of contributing to MSC treatment (52). MSCs may also stimulate angiogenesis in ischemic tissues, allowing for tissue and function regeneration, due to their capability to release angiogenic factors and drive endothelial differentiation. It has been determined that MSCs also perform neovascularization *via* a variety of angiogenic factors such as VEGF and angiopoietin1 (Ang1) (53).

Mesenchymal stem cell (MSC) has been examined in clinical trials in immune system disorders in the light of promising results after studying its immunomodulatory effects in a variety of disease models. It has also been reported that MSC transplantation, applied at an early stage, provides a therapeutic benefit in severe COVID-19 patients with anti-inflammatory and immunomodulatory effects (42, 43, 54). Despite the favorable effects of MSC being noted in clinical trials for autoimmune diseases, some investigations for a range of immune disorders, such as Diabetes mellitus, Systemic Lupus Erythematosus (SLE) did not reveal significant improvement in the symptoms of the disease (55). In this regard, with the improvement in the symptoms of the disease based on the NIHSS and RMA scores after the MSC treatment for stroke we acquired, our study may have intriguing results in the literature. Rikhtegar et al. reported that systemic or peripheral administration of MSCs is a safe avenue for stem cell transplantation, and intravenous administration of MSCs is functional for post-stroke recovery (56, 57). In a clinical trial conducted in 2013 to assess the safety, feasibility, and efficacy of mesenchymal cell transplantation, no deaths or adverse events were reported in patients who received MSCs intravenously where only one group of stroke patients indicated an increase in improvement. Consequently, intravenous stem cell treatment was reported to be both safe and viable (58). Jaillard et al. evaluated the safety, feasibility, and efficacy of MSC in subacute stroke and found no treatment benefit in patients who received intravenous MSC, except for sensorimotor behavioral results. MSC treatment, on the other hand, is safe and feasible and enables motor recovery through neuroplasticity, according to this study (59). Similarly, we demonstrated that intravenous UC-MSC transplantation had no negative side effects on stroke patients. Recent developments proved that restorative treatments based on neural repair, which promote neuroplasticity after stroke, have become significant. Neuronal recovery and plasticity after stroke begin early and last for several weeks. The acute response to damage occurs within the first hours, and the repair process begins in the first days following the stroke. When the endogenous recovery stabilizes weeks or months after the stroke, the repair process still enters a modifiable chronic phase (60). Patients who do not respond to established treatments, such as medicines, growth factors, or monoclonal antibodies may benefit from the novel therapy options we have introduced in our study (61).

In this study, we declared the non-invasive application of MSCs in the treatment of stroke as safe and beneficial. Herein we introduce the MSCs treatment for stroke, which supports novel therapies in other diseases requiring new treatment options.

Stroke became a global health problem with increasing morbidity and mortality rate, and therefore, it needs novel treatment options. Currently, there are no specific treatment options for stroke patients. In recent clinical studies, stem cell-based therapy is offering hope with beneficial results in stroke therapy. These studies and our study indicate that UC-MSC transplantation can significantly improve stroke patients’ quality of life and provide a basis for subsequent cell therapy investigations.

Our study is important in terms of demonstrating neural protection and recovery with anti-inflammatory and immunomodulation effects of MSCs for stroke treatment, and it provides critical preliminary data that will be important in guiding further research. In our study, the cohort was limited in number but sufficient to prove the safety and efficacy of the MSC treatment. However, further studies are required in large-scale groups to establish the beneficial treatment effects of UC-MSCs.

There are studies in the literature stating that stem cells are safe and effective, and positive results are obtained. However, we conducted this study to demonstrate that it improves patients’ clinical outcomes and to strengthen the information in the literature. Along with demonstrating the ability to cure, we also aimed to show how safe the standard technique we used with stem cells is. We recommend more extensive research and a controlled patient comparison using a standardized clinical protocol.

We discussed the outcomes of five patients in an inhomogeneous group that did not contraindicate supportive therapy. The treatment benefit group consisted of acute or chronic stroke patients. We determined that supportive care during the acute phase (4–6 weeks) positively influences outcomes, particularly in ischemic stroke patients. We recorded that supportive care had a significant impact on outcomes in patients with hemorrhagic stroke even after the patient had reached a stable period (after 4–6 weeks). In addition, we have noticed a positive effect on the outcomes when supportive treatment is applied even after 1 year in the chronic phase in ischemic stroke patients.

In the light of all these findings, we advise that the favorable impact of MSC supportive therapy on outcomes in patients with acute or chronic stroke should be investigated in larger cohort.

## Data availability statement

The original contributions presented in this study are included in the article/supplementary material, further inquiries can be directed to the corresponding author.

## Ethics statement

The studies involving human participants were reviewed and approved by Turkish Ministry of Health Stem Cell Commission with each family’s signed written consent form by the Declaration of Helsinki (Study design: Retrospective non-randomized clinical trial). The patients/participants provided their written informed consent to participate in this study. Written informed consent was obtained from the individual(s) for the publication of any potentially identifiable images or data included in this article.

## Author contributions

NE: conceptualization, developing the theory, and supervised the project. BK, OC, RA, GE, ZK, HS, ST, and CB: data curation, formal analysis, validation, and visualization. GG and NA: lab methodology. GB: project administration. NK: writing—original draft. NK and DE: writing—review and editing. NK, BK, BM, HS, and CB: investigation. All authors have read and approved the journal’s authorship agreement and the submitted version.
